# Intermittent hypoxia exacerbates tumor progression in a mouse model of lung cancer

**DOI:** 10.1038/s41598-020-58906-7

**Published:** 2020-02-05

**Authors:** Hye Seon Kang, Hee Young Kwon, In Kyoung Kim, Woo Ho Ban, Sei Won Kim, Hyeon Hui Kang, Chang Dong Yeo, Sang Haak Lee

**Affiliations:** 1 Division of Pulmonary, Critical Care and Sleep Medicine, Department of Internal Medicine, College of Medicine, The Catholic University of Korea, Seoul, Republic of Korea; 2 Cancer Research Institute, College of Medicine, The Catholic University of Korea, Seoul, Republic of Korea

**Keywords:** Lung cancer, Cell growth

## Abstract

The purpose of this study was to evaluate whether obstructive sleep apnea (OSA)-related chronic intermittent hypoxia (CIH) influences lung cancer progression and to elucidate the associated mechanisms in a mouse model of lung cancer. C57/BL6 mice in a CIH group were exposed to intermittent hypoxia for two weeks after tumor induction and compared with control mice (room air). Hypoxia inducible factor 1α (HIF-1α), vascular endothelial growth factor (VEGF) and metastasis-related matrix metalloproteinases (MMP) were measured. The expression levels of several hypoxia-related pathway proteins including HIF-1α, Wnt/ß-catenin, the nuclear factor erythroid 2-related factor 2 (Nrf2) and mammalian target of rapamycin-ERK were measured by western blot. The number (P < 0.01) and volume (P < 0.05) of tumors were increased in the CIH group. The activity of MMP-2 was enhanced after CIH treatment. The level of VEGF was increased significantly in the CIH group (p < 0.05). ß-catenin and Nrf2 were translocated to the nucleus and the levels of downstream effectors of Wnt/ß-catenin signaling increased after IH exposure. CIH enhanced proliferative and migratory properties of tumors in a mouse model of lung cancer. ß-catenin and Nrf2 appeared to be crucial mediators of tumor growth.

## Introduction

Obstructive sleep apnea (OSA) is characterized by recurrent occlusion of the upper airway during sleep leading to chronic intermittent hypoxia (CIH). OSA is a highly prevalent sleep disorder affecting at least 3% to 7% of the adult population^1^. OSA has been shown to be associated with morbidities including metabolic syndrome, systemic hypertension, pulmonary vascular disease, ischemic heart disease and congestive heart failure^2^. In addition, OSA-related CIH has been suggested to affect tumor development and progression^3^.

OSA has been implicated in the increasing incidence of certain types of solid malignancies. In a Spanish cohort, increased overnight hypoxia as a surrogate of OSA severity was associated with increased cancer incidence in selected populations^4–6^. Also, OSA was associated with increased cancer mortality in a community-based sample^7^.

The association between OSA and lung cancer has been evaluated. OSA-related CIH induced resistance to apoptosis and increased metastasis in lung cancer cells, in a manner related to hypoxia inducible factor 1α (HIF-1α)^8^. The role of immune cells has been suggested as a potential mechanism linking OSA and cancer. Almendros *et al*. demonstrated that CIH induced changes in host immune responses are related to adverse cancer outcomes. CIH increases the mobilization of tumor-associated macrophages (TAMs) into tumors, and induces macrophages to transform from an anti-tumor phenotype (M1) to a tumor-promoting phenotype (M2)^9^. Cyclooxygenase-2 inhibited IH-induced M2 polarization of TAMs and prevented IH-induced adverse tumor outcomes in a mouse model of sleep apnea^10^. OSA-related CIH altered CD8^+^ T-cells in combination with changes in the tumor microenvironment that enhanced malignant tumor properties^11^.

Studies have focused on CIH-related changes in the tumor microenvironment and immune cells such as T-cell lymphocytes or macrophages. However, except for HIF-1α, underlying transcriptional responses are poorly understood.

In the present study, we investigated whether CIH enhances lung cancer cell proliferation. We further identified tumor hypoxia-related mechanisms by evaluating multiple hypoxia-responsive genes regulating cancer progression and metastasis in a mouse model of lung cancer.

## Materials and Methods

### Experimental design and animal model

**Figure 1:**
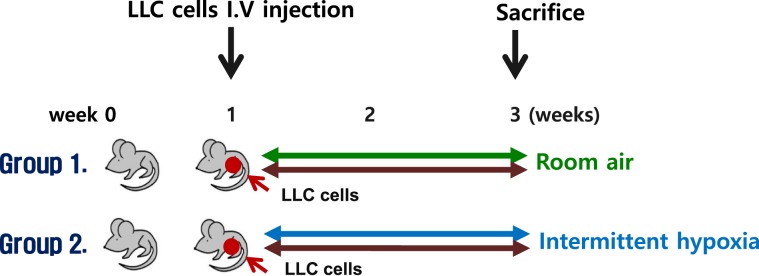
The animal experimental schedule. After tumor induction by tail vein injection of cells of the Lewis lung carcinoma line, the mice of the CIH group were exposed to intermittent hypoxia-inducing conditions for 2 weeks, while those of the control group breathed room air. At 3 weeks, the mice were sacrified and cancer growth was measured. LLC: Lewis lung carcinoma.

The experimental design is illustrated in Fig. 1. Seven-to-eight week-old male, C57BL/6J mice were purchased from Daehan Biolink (Chungcheongbuk-do, Korea). After 1 week of acclimation, the mice injected with Lewis lung carcinoma (LLC) cell lines through the tail vein. Each of the groups was further divided into two groups: (1) normoxia controls and (2) CIH. In each group, the number of mice was 8. Mice were exposed to air containing approximately 7 ± 0.5% fraction of inspired O_2_ (FiO_2_), 20 episodes/h, 8 h during daytime (CIH group) or room air (control group) for 2 weeks. The oxygen level was measured with a ProOx 110 analyzer (BioSpherix, Redfield, NY, USA). Diet and drinking water were provided *ad libitum*. All animals were maintained in a pathogen-free environment at room temperature (20 ± 2 °C) at a relative humidity of 50 ± 10% with ≥10–15 air changes/h under an alternating 12-h/12-h light/dark cycle. Body weight was monitored once per week for each mouse. This study was approved by the Ethical Committee on Animal Experiments of the Catholic University of Korea (SPH-20181123-01). All experimental animal protocols were approved by the Animal Subjects Committee at the Catholic University of Korea, in accordance with Article 14 of the Korean Animal Protection Law. The use of animals in the study complied with Article 13 of the Korean Animal Protection Law and relevant institutional policies.

### Lung tumor analysis and evaluation

After sacrifice, the number of lung tumor nodules was counted and the tumor volume was calculated. Tumors were dissected from the mice and measured with a Thorpe caliper. Nodules <1.5 mm in diameter were measured using the naked eye. Nodules ≤0.5 mm in diameter were microscopically categorized by diameter. Tumor volume was calculated using the following formula: (d^1^ × d^2^ × d^3^) × 0.5236, where d^n^ represents the three orthogonal diameter measurements. Hematoxylin and eosin (H&E)-stained slides of lung tissues were scanned with a Panoramic MIDI slide scanner (3DHISTECH Ltd., Budapest, Hungary).

### Bronchoalveolar lavage (BAL) fluid acquisition and analysis

After sacrifice, bronchoalveolar lavage (BAL) was performed by washing the airways twice with PBS from a 23 G needle syringe connected to a tracheal cannula. The total number of cells in the BAL fluid was determined by counting in a hematocytometer. To obtain the differential inflammatory cell count, BAL fluid was processed by cytospin (5 min at 750 × g) onto microscope slides and stained with Diff-Quik (Sysmex, Tokyo, Japan). The percentages of cells were obtained by counting at least 400 macrophages, lymphocytes, neutrophils or eosinophils on randomly selected areas of the slide using light microscopy.

### Histopathology and immunohistochemistry (IHC)

After sacrifice, the trachea was exposed and the left lung in each group were inflated with a 10% buffered-formalin solution. Lung tissues were fixed overnight and then routinely processed for histological inclusion in paraffin. Deparaffinized tissue sections were subjected to H&E staining for histological examination. IHC was performed on 3 μm thick section of formalin-fixed, paraffin-embedded tissue. Tissue sections were quenched for endogenous peroxidase and placed in an antigen retrieval solution(10 mM citrate buffer, pH 6.0) for 15 min at 95 °C in a steamer. Ki-67 (abcam; ab15580, UK) or CD31 (abcam; ab28364) was applied to the sections at dilutions of 1:100, and incubated overnight at 4 °C. Staining was developed with Diaminobezidine Chromogen (DAB), slides were counterstained with Harris hematoxylin.

### Scoring of Ki-67 and CD31 protein expression

Ki-67 scoring-sections were examined under 400 magnification by two investigators who were blinded to the histopathological data, and scored based on the extent of nuclei immunostaining (the percentage of positive nuclei). We modified the scoring system slightly as follows: 0 for <5% positive cells, 1 for 5–15% positive cells, 2 for 16–30% positive cells, 3 for 31–50% positive cells, 4 for 51–75% positive cells, and 5 for >75% positive cells. Five hundred cells were counted and calculated the average percentage manually in the Ki-67 positive tumor cells in the three microphotos^12^. The CD31-stained sections were semi-quantitatively scored as (+), (++), or (+++) in five visual microscopic fields selected randomly by endothelial cells of blood vessels per sample and were expressed as mean per sample. These were assigned arbitrary numerical values of 2, 4, and 8, respectively^13^.

### Enzyme-linked immunosorbent assay

HIF-1α (Cell Biolabs, Inc., San Diego, CA, USA) and vascular endothelial growth factor (VEGF; R&D systems, Minneapolis, MN, USA) levels were measured by ELISA according to the manufacturer’s instructions. The amounts of mouse HIF-1α, or VEGF present were determined by known concentrations, with a standard curve, respectively. Plates were read at 450 nm in a microplate reader.

### Western blotting and zymography

Lung homogenate proteins were isolated using a Nuclear/Cytosol fractionation kit (Thermo Fisher Scientific Inc, Bartlesville, OK, USA). Equal amounts of lysate protein were separated by 8–15% SDS-PAGE and transferred to PVDF membranes. Membranes were blocked in 5% skim milk. They were then incubated with primary antibodies against Proliferating cell nuclear antigen (PCNA), Glucose transporter 1 (GLUT1), (Santa Cruz Biotechnology, Inc., TX, USA.), Carbonic anhydrase 9 (CA9) (GeneTex, Inc. CA, USA.), c-Myc, cyclin D1, cyclin-dependent kinase 4 (CDK4), survivin, p21, HIF-1α, VEGF, Bcl2, mammalian target of rapamycin (mTOR), ERK1/2, β-catenin, the nuclear factor erythroid 2-related factor 2 (Nrf2), or β-actin (Cell Signaling Technology, Inc., Danvers, MA, USA) at a 1:1,000 dilution at 4 °C overnight. Target proteins were detected using the enhanced chemiluminescence method (Bio-Rad, Hercules, CA, USA). Signal detection was performed using ImageQuant LAS 500 Imaging System (GE Healthcare, Life Sciences, Korea). Collagenolytic activities in serum were measured by gelatin zymography. The gels were stained with Coomassie Brilliant Blue R-250 and then destained with methanol-acetic acid-water.

### Data analysis

The data were analyzed using one-way analysis of variance followed by Tukey’s multiple comparison test or two-way ANOVA followed by Bonferroni multiple comparisons post-test using GraphPad Prism version 7.00 (GraphPad Software, San Diego, CA, USA). All data are expressed as means ± standard deviation (SD), and in all cases, differences were considered statistically significant at *P* < 0.05.

## Results

### CIH promotes accelerated tumor growth with increasing numbers and volumes

**Figure 2:**
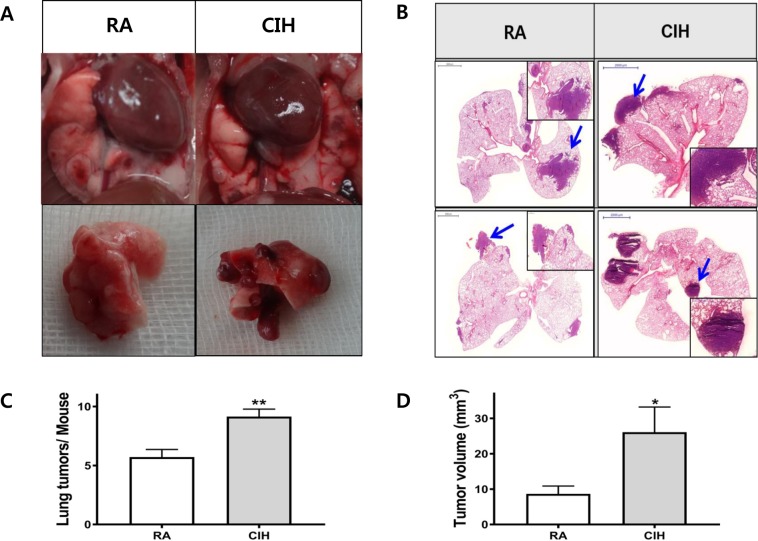
Effects of CIH on the histopathologic changes and tumor growth in lung tissue in a mouse model of LLC. (**A**) Representative images of gross lung examination in LLC mice. (**B**) Photographs of the dissected tumors and representative images of hematoxylin and eosin stained lung sections. The two top panels show images of whole lung sections and higher magnification of the same samples (magnification, x400). Lung tumor development was evaluated according to (**C**) the number of tumors and (**D**) the tumor volume in each group at the study endpoint. The data represent the mean ± SD. *p < 0.05, **p < 0.01 compared with RA controls. CIH: chronic intermittent hypoxia; LLC: Lewis lung carcinoma; RA: room air; SD: standard deviation.

Gross findings showed that CIH enhanced tumor volumes and numbers (Fig. 2A) Tumor cells from CIH mice displayed a greater ability to colonize the lung and grow into overt tumor loci. The lungs of mice exposed to CIH contained more and larger metastases compared to controls (Fig. 2B). CIH exposure promoted faster tumor growth, increasing tumor number and volume (9.14 ± 1.68 and 26.09 ± 17.46 cm^3^) compared controls (5.71 ± 1.70, *P* < 0.01; 8.66 ± 5.87 cm^3^, *P* < 0.05) (Fig. 2C,D).

### CIH did not induce changes in body weight and inflammatory cells

**Figure 3:**
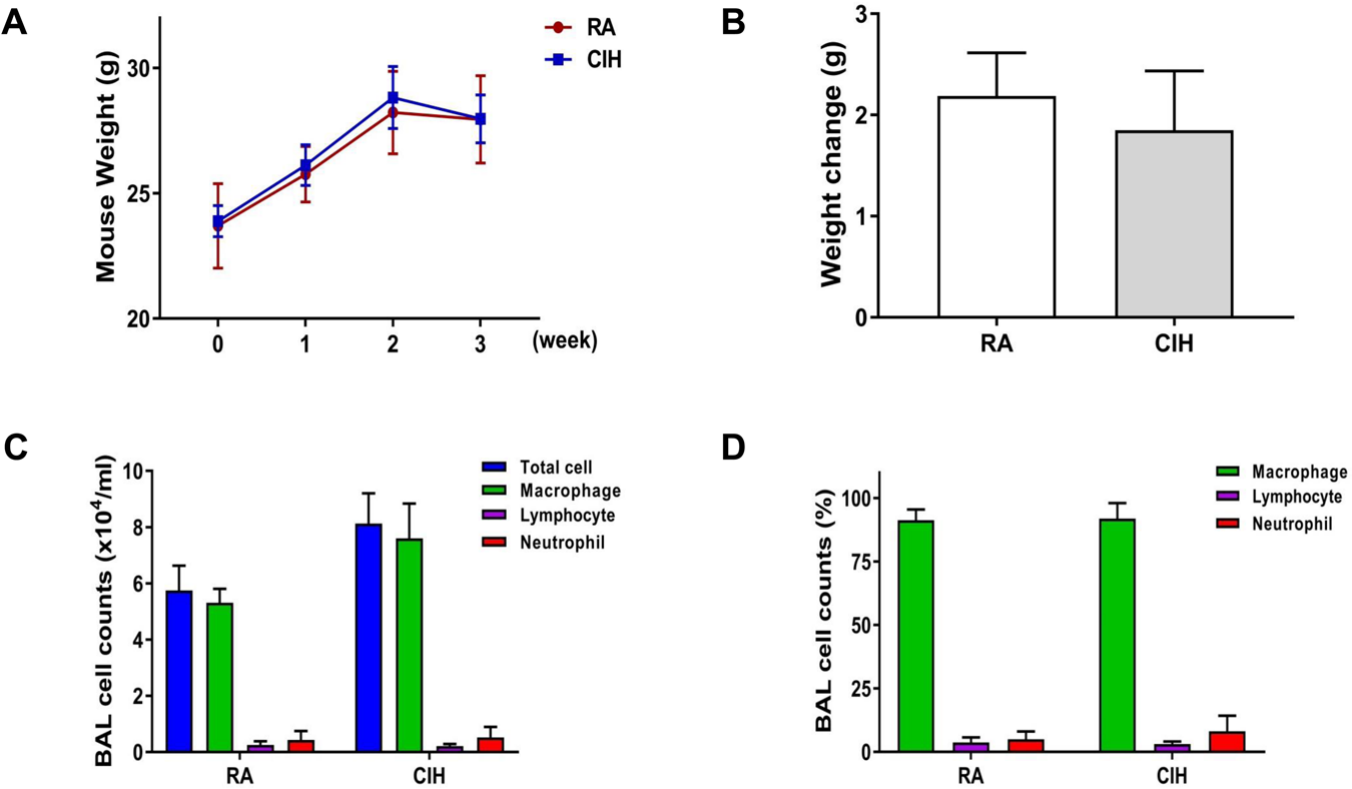
Effects of CIH on body weight and number of inflammatory cells in BAL fluid in a LLC mouse model. (**A**) Body weight (BW) of mice in the RA or CIH groups. BW was measured weekly at a regular time. (**B**) Comparison of BW gain in mice (ΔBW = BW 3 weeks – BW 1 week). BAL cells were isolated and total and differential cells were (**C**) counted or (**D**) expressed in percentages to analyze inflammatory cells. The results are the means ± SD. BAL: bronchoalveolar lavage; CIH: chronic intermittent hypoxia; LLC: Lewis lung carcinoma; RA: room air; SD: standard deviation.

The body weights of mice with lung tumors were measured weekly at a regular time. Body weight changes (ΔBW = BW 3 weeks – BW 1 week) were not different between the control and CIH groups (Fig. 3A,B). Inflammatory cells were isolated from BAL fluid, and total and differential counts were analyzed. The counts and percentages of inflammatory cells including macrophages, lymphocytes, and neutrophils were not different between the control and CIH groups (Fig. 3C,D).

### CIH enhances MMP-2 activity

**Figure 4:**
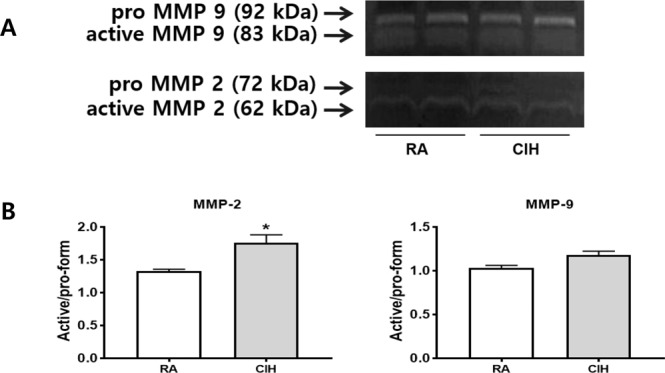
Effects of CIH on the expression of MMP-2 and MMP-9. (**A**) Gelatin zymography analysis for MMP-2 and MMP-9 in serum. (**B**) Quantitative analysis of the MMP-2 and MMP-9 activation ratios in serum from the RA and CIH groups. The values represent means ± SD. ^*^p < 0.05 compared with RA controls. CIH: chronic intermittent hypoxia; MMP: matrix metalloproteinase; RA: room air; SD: standard deviation.

The gelatinases MMP-2 (gelatinase A) and MMP-9 (gelatinase B) are two members of the MMP family that are known to be associated with tumor invasion, metastasis and progression^14^. The activities of MMP-2 and MMP-9 were examined by gelatin zymography. The analysis of MMP-9 revealed no statistical differences between control and CIH groups. However, MMP-2 activity significantly increased after CIH treatment. (Fig. 4A) The MMP-9 and MMP-2 activation ratios in the sera were measured. The activation ratio of MMP-2 (1.76 ± 0.21 vs. 1.33 ± 0.05, *P* = 0.005) was significantly higher in the CIH group compared to the control group, respectively. That of MMP-9 (1.18 ± 0.08 vs. 1.03 ± 0.05, *P* = 0.0531), however, was not different between the two groups (Fig. 4B).

### CIH induces the expression of Ki-67 and CD31

**Figure 5:**
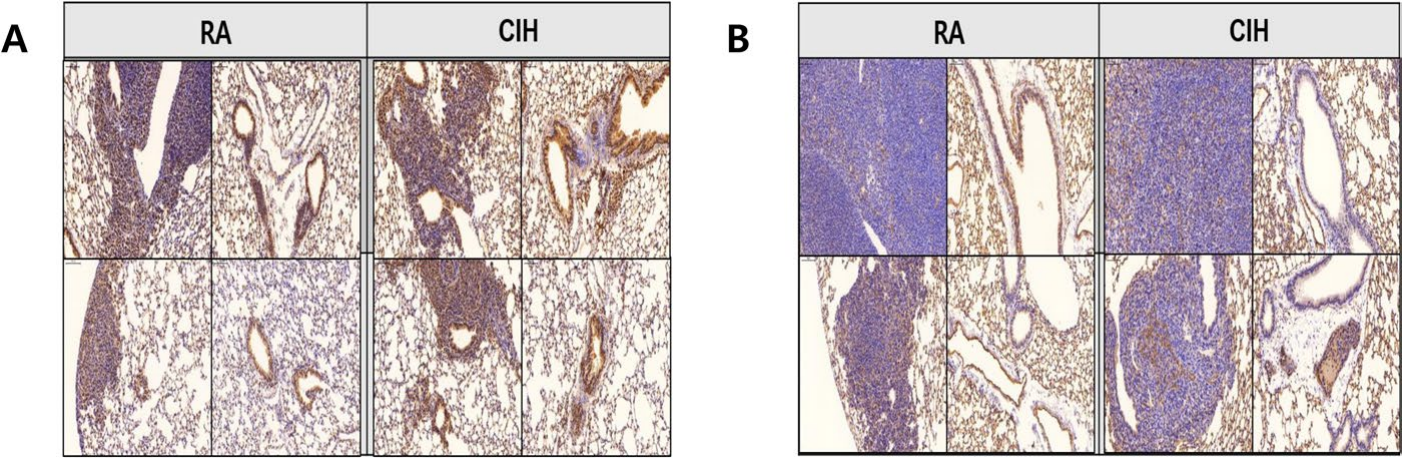
Effects of CIH on the proliferation and angiogenesis in a mouse model of LLC. The representative images of immunohistochemistry of (**A**) Ki-67 and (**B**) CD31 in lung sections (magnification, x200). CIH: chronic intermittent hypoxia; LLC: Lewis lung carcinoma.

Ki-67 is a proliferating index and CD31 is an endothelial cell and angiogenic biomarker^15,16^. IHC staining was performed to investigate the effect of CIH on Ki-67 and CD31 expression in mice with lung tumors. In the CIH group, scoring of Ki-67 expression based on average percentage was significantly higher than that in the controls. (4.25 ± 0.50 vs. 3.08 ± 0.74, *P* < 0.05) When the tissue sections were semi-quantitatively ranked on the basis of intensity of staining exhibited by the endothelial cells in blood vessels, the tissue sections from CIH group received a significantly greater mean score than controls (5.10 ± 0.50 vs. 3.30 ± 1.10, *P* < 0.05) (Fig. 5A,B).

### CIH does not induce HIF-1α expression but does induce VEGF expression

**Figure 6:**
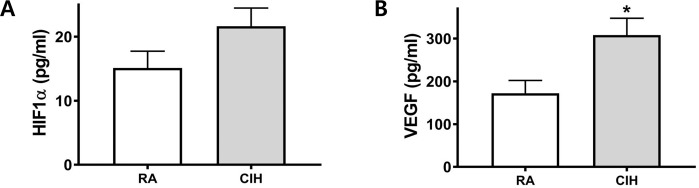
Effects of CIH on the expression of HIF-1α and VEGF in a mouse model of LLC. The levels of (**A**) HIF-1α (nuclear fraction) and (**B**) VEGF were measured in the lung homogenate using ELISA. ^*^p < 0.05 compared with RA controls. CIH: chronic intermittent hypoxia; HIF-1α: hypoxia inducible factor 1α; LLC: Lewis lung carcinoma; RA: room air; VEGF: vascular endothelial growth factor.

ELISA assays were performed to investigate the effect of CIH on HIF-1α and VEGF expression in mice with lung tumors. HIF-1α and VEGF expression were measured in lung homogenate after isolating nucleus and total lung homogenate, respectively. In the CIH group, the VEGF expression level was significantly higher than that in the controls (308.09 ± 104.29 vs. 172.00 ± 90.62 pg/mL, *P* < 0.05). The CIH group did not show a significant change in HIF-1α expression compared to the controls (Fig. 6A,B).

### CIH induces the translocation of ß-catenin and Nrf2 into the nucleus

**Figure 7:**
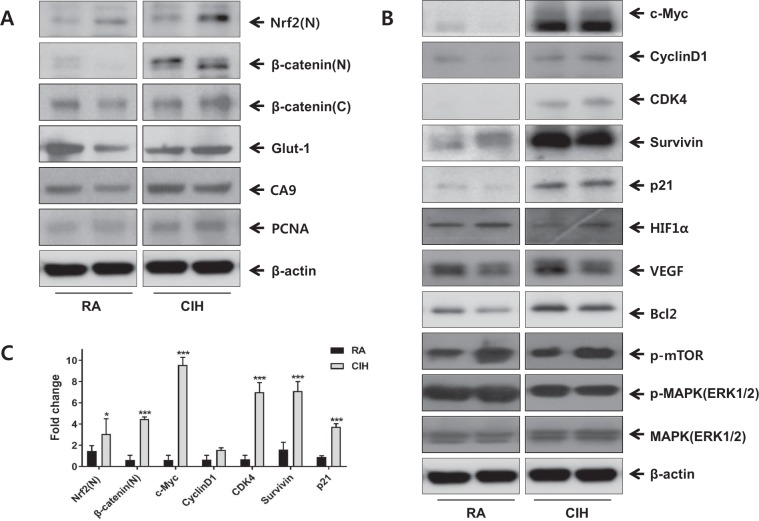
Effect of CIH on the protein levels of several hypoxia-related genes in a mouse model of LLC. Protein levels were examined in total protein by western blotting. The representative indicators were measured in the lung homogenate (N:nucleus, C:cytoplasm, other:Total). β-actin was used as a control. ^***^p < 0.001, ^**^p < 0.01 compared with RA controls. RA: room air; CIH: chronic intermittent hypoxia; LLC: Lewis lung carcinoma; Nrf2: the nuclear factor erythroid 2-related factor 2; Glut-1: glucose transporter 1; CA9: carbonic anhydrase IX; PCNA: proliferating cell nuclear antigen; CDK4: cyclin-dependent kinase 4; HIF1a: hypoxia inducible factor 1α; VEGF: vascular endothelial growth factor; mTOR: mammalian target of rapamycin.

ß-catenin is the major transcriptional co-activator in the Wnt signaling pathway, and mutation of ß-catenin is associated with cell proliferation and carcinogenesis. We examined whether CIH affected the levels of ß-catenin and Nrf2 proteins in the mice with lung tumors. A western blot assay showed that CIH upregulated the protein level of ß-catenin approximately 7.32-fold in nuclear fractions (*P* < 0.001). Nrf2 protein significantly increased approximately 2.12-fold after CIH treatment when compared to controls (*P* < 0.05) (Fig. 7A,C).

### CIH induces the expression of CA9 and PCNA

To determine whether CIH affects other hypoxic hypoxia marker, CA9 and GLUT1. A western blot assay showed that CIH upregulated the protein level of CA9 in the mice with lung tumors. However, GLUT1 was not enhanced after CIH treatment. The expression of PCNA, which have distinct roles in cell growth, was enhanced after CIH treatment compared to the control in western blot (Fig. 7A).

### CIH increases c-Myc, Cyclin D1, CDK4, survivin and p21 expression

To determine whether CIH affects the Wnt/ß-catenin signaling pathway, we examined the downstream effectors of the Wnt/ß-catenin pathway including c-Myc, cyclin D1, CDK4, p21 and survivin using western blots^17–24^. The lung homogenates from the CIH treated mice with lung tumors showed significant increases in the protein levels of c-Myc, CDK4, p21 and survivin. The protein expression of cyclin D1 increased approximately 2.43-fold even though there is no statistically significance (Fig. 7B,C). Mammalian target of rapamycin (mTOR) signaling, another potent pathway responsive to hypoxia, did not change with CIH.

## Discussion

In this study, we found that OSA-related CIH accelerated tumor development including proliferative and metastatic properties, in a mouse model of lung cancer. When the underlying molecular mechanisms were examined, ß-catenin and Nrf2 were translocated to the nucleus, and the levels of downstream effectors of Wnt/ß-catenin signaling (i.e., c-Myc, cyclin D1, CDK4, p21, VEGF and surviving) increased in CIH-exposed mice with lung tumors. However, the expression levels of HIF-1α and mTOR, two representative hypoxia sensors, did not change under those conditions.

Other studies have demonstrated that CIH is associated with accelerated tumor growth, migration and metastasis in different types of malignancies including malignant melanoma, breast cancer and prostate cancer^25–29^. Our results were consistent with those studies. The numbers and volumes of tumors were greater in mice exposed to CIH than those exposed to normoxia. MMP-2 activity and the expression of PCNA, Ki-67 and CD31 which are associated with cancer growth, invasion, and migration, increased after CIH treatment.

Chronic hypoxia is recognized as an important feature of the intra-tumoral environment and is associated with induction of tumor growth and metastasis^29^. In contrast with chronic hypoxia, OSA-related CIH has a unique physiologic character with cycles of post-hypoxic re-oxygenation^27^. The effects of chronic tumor hypoxia, characterized by limited blood and oxygen supply, have been well studied, but the effects of the cyclic changes of OSA-induced hypoxia on cancer biology are not well known^30^.

Small animal models have demonstrated an important role for systemic CIH in tumor growth and metastasis, but the underlying mechanisms are poorly understood. There are various postulated mechanisms by which CIH may affect tumor biology. General mechanisms that are thought to play a role include oxygen-sensing pathways and oxidative stress. One proposed mechanism involves CIH induced activation of many transcriptional factors, such as HIF-1α, activator protein-1, nuclear factor kappa-light-chain-enhancer of activated B cells and Nrf2, and expression of specific genes associated with tumorigenesis^30^. The immune response is another important component of the tumor microenvironment, and immune cell activities are suggested as potential mechanisms linking OSA and cancer^10^.

In lung cancer, CIH induces cancer cell resistance to radiotherapy and apoptosis. Such changes appeared to be related to HIF-1α^8^. CIH increased VEGF expression and microvessel density in a mouse model of OSA^31^. CIH-induced alterations in tumor-associated macrophages (TAMs) were associated with adverse cancer outcomes in a mouse model of lung cancer^9^. CIH is known to be associated with elevated oxidative stress and inflammation. Nevertheless, CIH-related and oxidative stress related transcriptional responses are poorly understood in lung cancer. Several signaling pathways are activated in cancer cells to adjust to hypoxic environments. Although HIF-1α constitutes a potent hypoxic response mechanism, other pathways such as the phosphatidylinositol 3-kinases (PI3K)-Akt-mTOR pathway and Wnt/ß–catenin also participate in the adaptation of cancer cells to hypoxic conditions. Here, we hypothesized that CIH-induced oxidative stress and the response to hypoxia may play important roles in lung cancer progression and metastasis, and we investigated oxidative stress-related transcriptional factors in a mouse model of lung cancer.

Activation of Wnt/ß-catenin signaling can induce cell proliferation and invasion, which are critical in promoting carcinogenesis^32^. During tumorigenesis, accumulation of ß-catenin in the cytoplasm is recognized as the hallmark of the Wnt signaling pathway^33^. Wnt signaling induces ß-catenin translocation to the nucleus to form the ß-catenin/T-cell factor transcriptional activator which activates a number of target genes including survivin, cyclin D1, c-Myc and VEGF. The elevated expression of Wnt/ß-catenin downstream effectors then enhances cell proliferation^34–36^. Sustained-hypoxia-induced cancer progression via Wnt/ß-catenin signaling has been reported in various cancers including lung, liver, kidney and bladder^37–41^. However, interactions between OSA-related IH and Wnt/ß-catenin signaling in lung cancer have rarely been reported.

In this study, ß-catenin was translocated to the nucleus after CIH treatment. The protein expression levels of Wnt target genes including cyclin D1, VEGF, c-myc and survivin were enhanced after CIH treatment in a mouse model of lung cancer^21–24^. Cyclin D1 regulates the cell cycle by controlling G1/S transition, and the up-regulation of cyclin D1 is related to the development of various cancers^42,43^. C-Myc is an oncogenic transcription factor, recognizing E-box and related sequences in the promoters of target genes. An increased level of c-Myc is associated with tumor metastasis and invasion^44,45^. Survivin is highly expressed in most human cancers, and aberrant survivin expression is associated with cancer cell proliferation, progression and angiogenesis. Survivin is one of the members of the inhibitor of apoptosis protein family, which is important in regulating cell division and inhibiting apoptosis by blocking caspase activation^46^. In our study, CIH induced the expression of Wnt/ß-catenin signaling related genes, as well as the translocation of ß-catenin into the nucleus.

Furthermore, CIH increased Nrf2 expression in mouse lung tumor tissue in our study. Nrf2 is a nuclear transcription factor that controls the expression of cytoprotective genes in response to oxidative stress. Under oxidative stress, Nrf2 is stabilized and translocated to the nucleus, where it binds to an antioxidant response element, which in turn leads to coordinated activation of gene expression. Induction of this adaptive response confers up-regulation of defensive enzyme levels and reduces the cytotoxic effect of reactive oxygen species^47^.

A role for Nrf2 in the response to CIH has been suggested, although opposing effects have been observed depending on the tissue type^3,48^. Further, Nrf2 can prevent or support cancer progression depending on the cell types. Nrf2 has been considered as a tumor suppressor because of its cytoprotective functions against exogenous or endogenous insults, including xenobiotics and oxidative stress^49^. Nrf2 prevents carcinogens from reaching target sites, inhibits parent molecules undergoing metabolic activation, and prevents carcinogenic species from interacting with crucial cellular molecules^50^. However, hyper-activation of the Nrf2 pathway induced by gain-of-function mutations creates an environment that favors the survival of malignant cells^51^. Nrf2-dependent creation of a more favorable intracellular environment for the survival of tumor cells may promote tumorigenesis^52^. Enhanced Nrf2 activity is also associated with resistance of cancer cells to chemotherapy^53,54^.

In our study, other hypoxic sensors, such as CA9 and Glut1 were investigated. The expression of CA9 was enhanced, but that of Glut-1 was not changed after CIH treatment. CA9, an intracellular pH regulator that promotes cell survival, and functions as cellular response to hypoxia^55,56^. CA9 is not detected in most normal tissues, but its expression in various cancers indicates hypoxic tumors and poor treatment response^57^. Glut1 is one of hypoxia biomarkers and associated with poor prognosis in various cancers^58–60^. Glut1 is HIF-1 target genes and enhanced in cells exposed to intermittent hypoxia in colon cancer^61^. In our study, it might be explained that GLUT1, target gene of HIF1, is not expressed because HIF1 is not enhanced after CIH treatment.

Interesting observation of the current study is that HIF-1α, an important transcriptional factor related with tumor apoptosis and metastasis in hypoxic tumor environment, was not changed with CIH. This finding could be explained that high expression of HIF-1α in normoxic conditions in lung cancer and rapid degradation of HIF-1α on reoxygenation after hypoxia. Whereas hypoxia is associated with decreased degradation of HIF-1α and increased HIF-1α levels, growth factors, cytokines and other signaling molecules stimulate HIF-1α synthesis via activation of the PI3K or mitogen-activated protein kinase pathways^62^. In previous studies, basal level of HIF-1α expression is high in lung cancer mouse model in normoxic condition even though the level of HIF-1α is increased in hypoxic conditions^63–65^. Also, it is assumed that HIF-1α and HIF-2α are activated differentially depending on the duration of hypoxia^66^. In breast cancer cells, acute hypoxia increased HIF-1α expression, while chronic hypoxia continuously enhanced HIF-2α expression and induced the resistance of breast cancer cells to chemotherapy^67^. HIF-1α protein levels typically peak around 4–8 h and continuously decrease thereafter and are undetectable around 18–24 h; while HIF-2α levels are stabilized relatively later and tend to play a key role during chronic hypoxia (24–72 h)^66,68^. This phenomenon is explained that HIF-1α in hypoxia leads to accelerated degradation on reoxygenation after hypoxia by induction of HIF-α-prolyl-4-hydroxylases^69^. Little studies conducted about the effects of OSA-related CIH on different types of HIF expression in a mouse model of lung cancer, further study is needed to fully understand exact mechanisms.

In our study, CIH induced Nrf2 expression as well as Wnt/ß-catenin signaling in a mouse model of lung cancer. In a previous study, taxifolin, which is a natural compound found in *Pinus roxburghii*, elicitd anti-carcinogenic potential activity through Nrf2-mediated anti-inflammation and Wnt/ß-catenin inhibition in colon cancer^70^. However, ß-catenin creates a protumorigenic environment by indirectly activating Nrf2 in hepatoblastoma cells. As mentioned above, Nrf2 plays a dual role in tumorigenesis. In the first study, Nrf2 activation reduced carcinogenesis by anti-inflammatory activity. However, in the latter case, Nrf2 activation due to oxidative stress favored the survival of malignant cells. Our study showed that CIH enhanced the expression of Nrf2 and Wnt/ß-catenin-related genes, implicating oxidative stress as a possible driving force for lung cancer progression.

Taken together, CIH induced cancer proliferation and metastasis in a mouse model of lung cancer. Our findings elucidated a novel regulatory function for CIH through activation of Wnt/ß-catenin signaling and Nrf2. These results suggest that CIH induced Wnt/ß-catenin signaling is a mediator of oncogenesis.
